# Supplementary material for: Surface ocean pH variations since 1689 CE and recent ocean acidification in the tropical South Pacific
Source: Nat Commun. 2018 Jun 29;9:2543. doi: 10.1038/s41467-018-04922-1 (PMC6026204; doi:10.1038/s41467-018-04922-1)
Supplement: Supplementary file 1 — Supplementary Information [file 41467_2018_4922_MOESM1_ESM.pdf]

Supplementary Information for

Surface ocean pH variations since 1689 CE and recent ocean acidification in the tropical south Pacific

Henry C. Wu[1,2,3*], Delphine Dissard[1], Eric Douville[2], Dominique Blamart[2], Louise Bordier[2], Aline Tribollet[1], Florence Le Cornec[1], Edwige Pons-Branchu[2], Arnaud Dapoigny[2], and Claire E. Lazareth[1]

[1] Institut de Recherche pour le Développement (IRD), Sorbonne Universités (UPMC Université Paris 06, CNRS, MNHN), UMR LOCEAN/IPSL, IRD DR Ile-de-France, 32 Avenue Henri Varagnat, F-93143 Bondy, France.

[2] Laboratoire des Sciences du Climat et de l'Environnement, LSCE/IPSL, CEA-CNRS-UVSQ, Université Paris-Saclay, Bât. 12, Avenue de la Terrasse, F-91198 Gif-sur-Yvette, France.

[3] Now at Leibniz Centre for Tropical Marine Research (ZMT) GmbH, Fahrenheitstraße 6, D-28359 Bremen, Germany.

[*]Correspondence to: henry.wu@leibniz-zmt.de

This PDF file includes:
Supplementary Figures 1 to 12
Supplementary Tables 1 to 5
Supplementary References

Supplementary Figures.

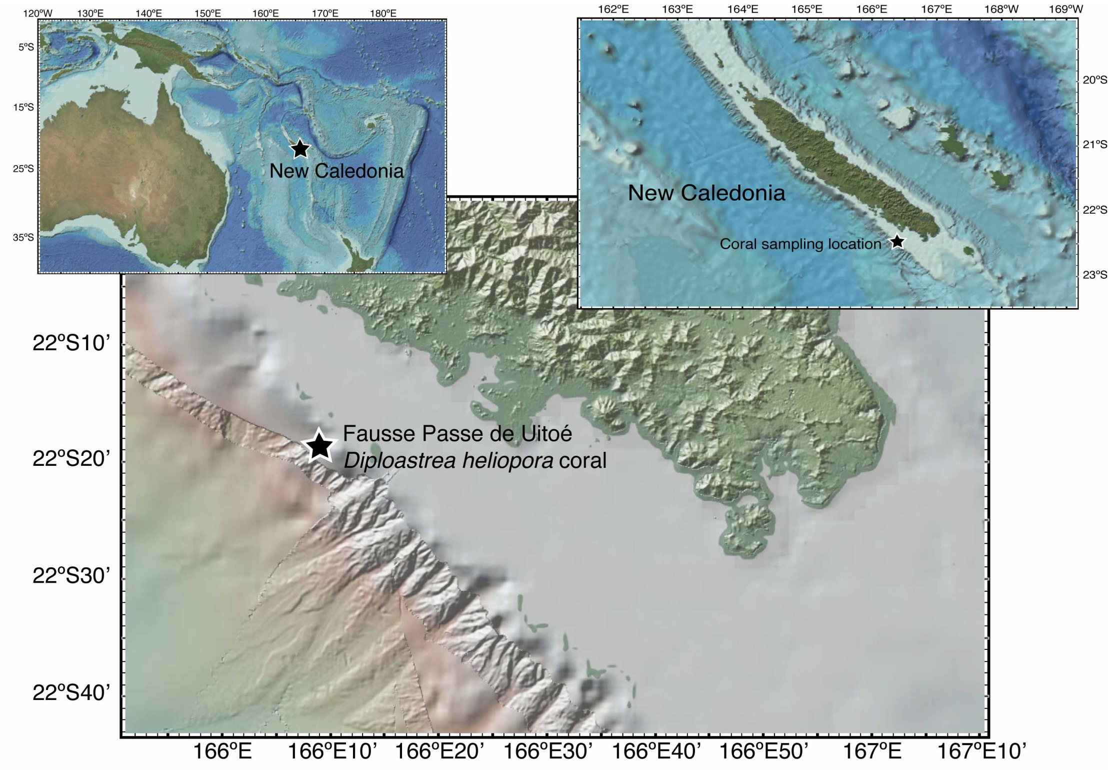

Supplementary Fig. 1. Research location.

Diploastrea heliopora coral core collection in 2015 at the Fausse Passe de Uitoé, New Caledonia (22º17'152 S, 166º10'992 E, Fig. 1). The collection area is outside of the shallow reef lagoon shown with the local bathymetry of the surrounding region. All maps generated from GeoMapApp (http://www.geomapapp.org) with the default basemap [1] and edited manually.

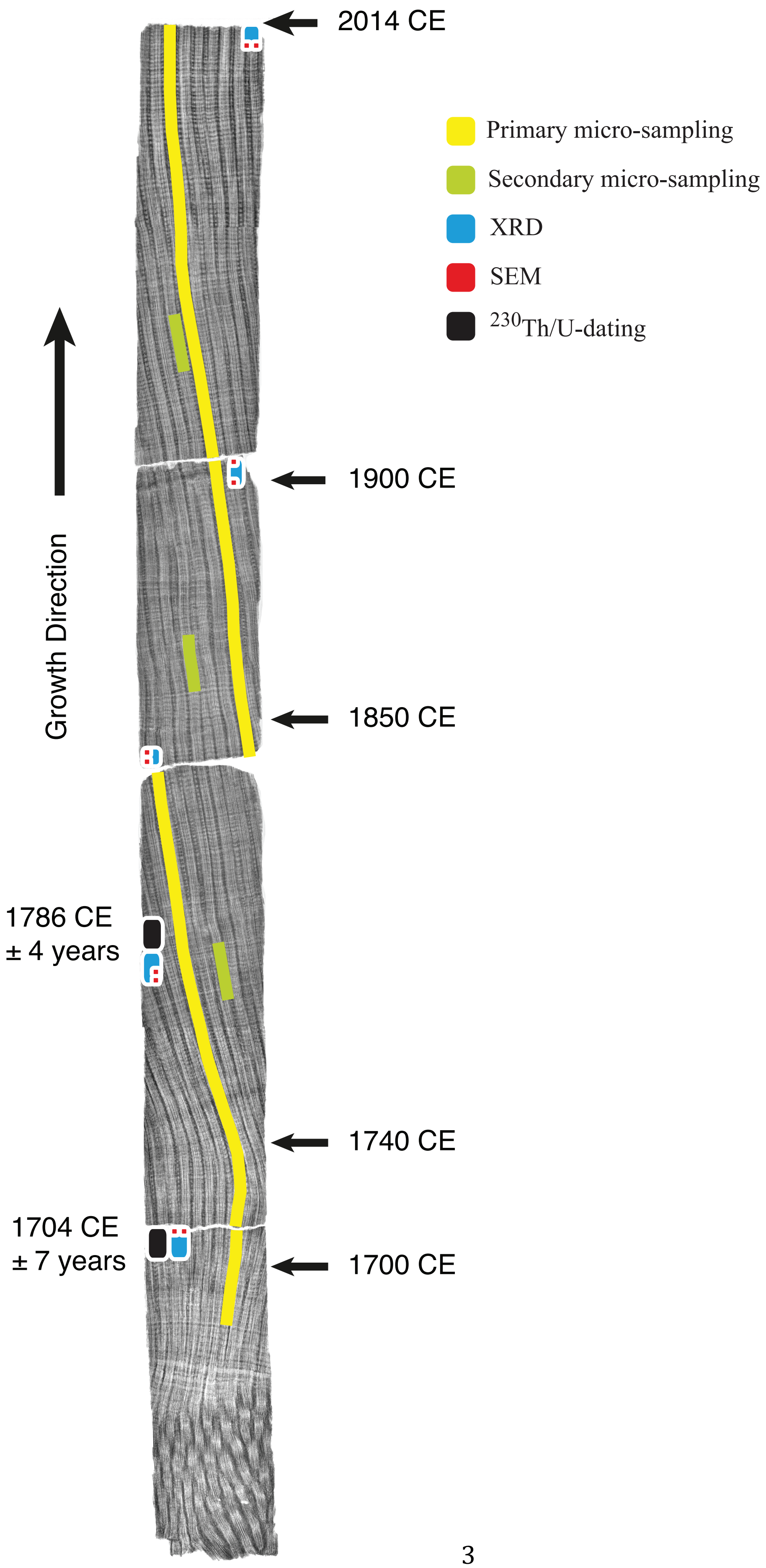
2014 CE
Primary micro-sampling
Secondary micro-sampling
XRD
SEM
230Th/U-dating
Growth Direction
1900 CE
1850 CE
1786 CE
± 4 years
1740 CE
1704 CE
± 7 years
1700 CE

Supplementary Fig. 2. ***Diploastrea heliopora*** **coral x-radiograph image.** The coral micro-sampling transects, microstructure analyses, and ^{230}Th/U-dating samples taken from the *D. heliopora* coral slabs are shown on the x-radiograph positive collage with markers for major years. The x-radiograph image depicts the perfect horizontal annual growth density banding of the *D. heliopora* coral relative to the vertical skeletal extension with primary (yellow) micro-sampling transect. The secondary (green) micro-sampling transects were completed in parallel to the primary transect at three independent 20-year intervals for intra-coral δ^{11}B reproducibility tests (Supplementary Fig. 8). Powder X-ray Diffraction (XRD) samples were taken at five different coral growth periods across the entire coral colony. Sub-samples of the coral skeleton were also taken next to the powder XRD samples and examined using Scanning Electron Microscopy (SEM; Supplementary Fig. 3). Two samples were removed from the coral slabs for ^{230}Th/U-dating and provided ages that are consistent with the density banding counting. These results confirm that the coral grew continuously over the period 1689-2014 CE.

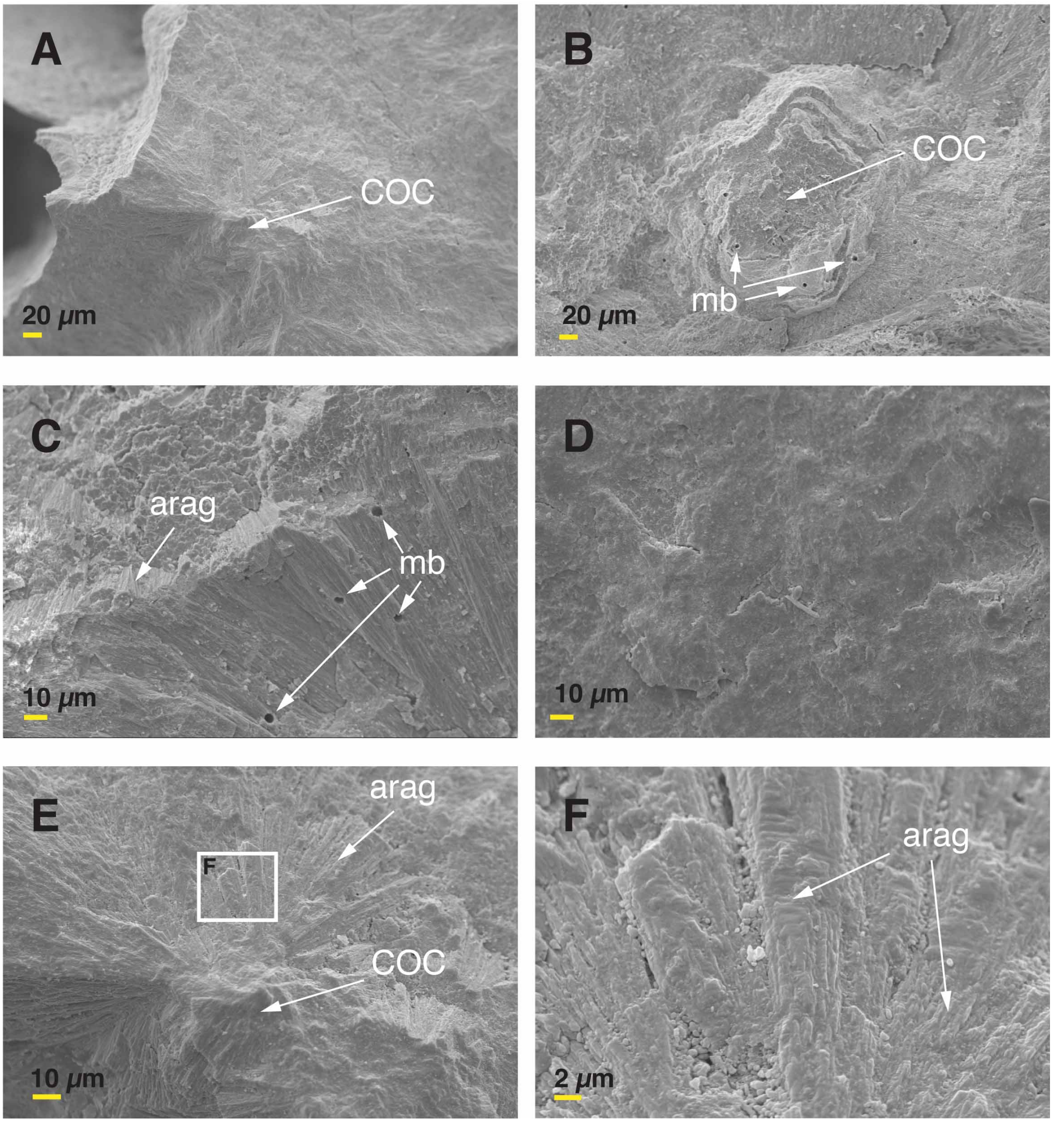

Supplementary Fig. 3. ***D. heliopora*** **coral Scanning Electron Microscopy images.**

The pristine nature of the *D. heliopora* coral microstructures was confirmed using Scanning Electron Microscopy (SEM) from five skeletal samples removed near the powder XRD analysis and distributed along the whole coral core. The skeletal samples were removed as close to the micro-sampling transect as possible (Supplementary Fig. 2). Clear centres of calcification (COC) with unaltered aragonite crystal (arag) were observed without signs of secondary aragonite, calcite infilling, or post-depositional dissolution or diagenetic alterations that attest the high quality of skeletal preservation. Some microboring (mb) excavations were observed throughout the coral core.

Coral: *Diploastrea heliopora*
Collection: Fausse Passe de Uitoé, New Caledonia (22º17’152 S, 166º10’992 E)
Total age: 1689-2014 CE

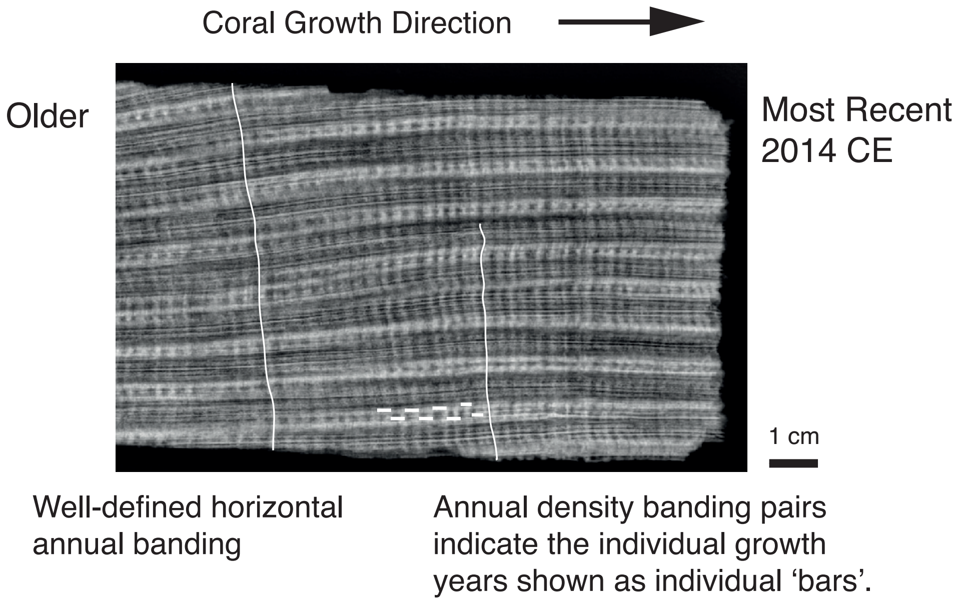

Supplementary Fig. 4. Detailed view of the *D. heliopora* density banding. The x-radiograph negative image depicts the detailed resolution view of the perfect horizontal annual growth density banding (thin white lines) of the *D. heliopora* coral relative to the vertical skeletal extension in the most recent portion of the coral core. Horizontal bars denote the individual years of annual density banding pairs.

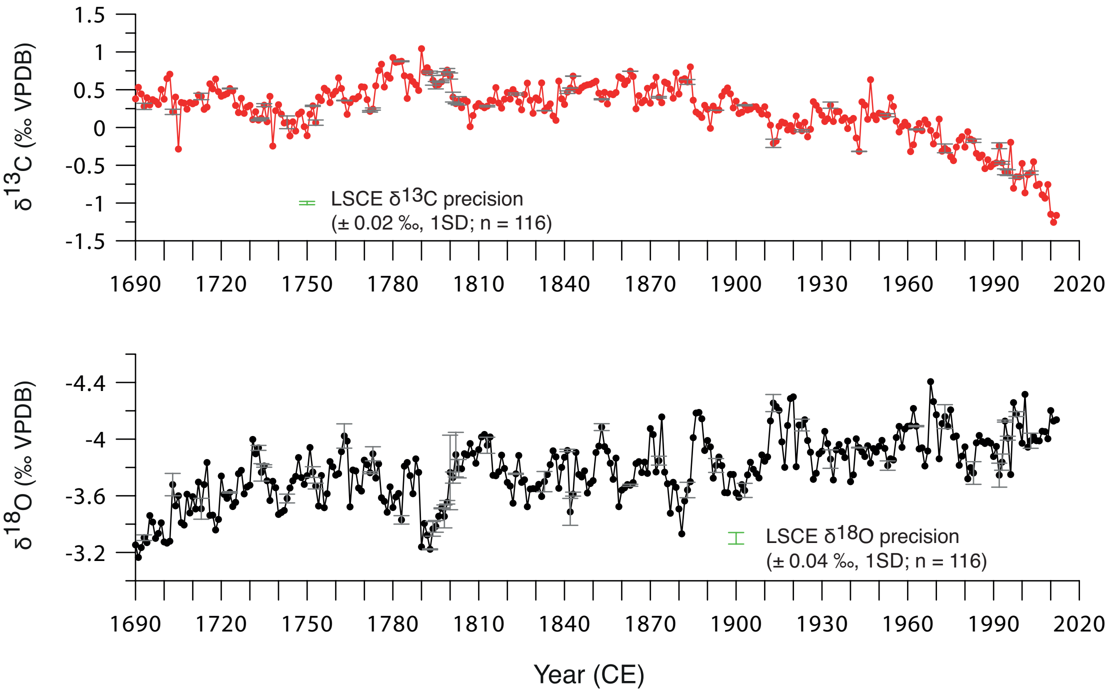

Supplementary Fig. 5. *D. heliopora* coral $\delta^{13}C$ and $\delta^{18}O$ time series. Annually-resolved coral $\delta^{13}C$ and $\delta^{18}O$ ratios are reported in ‰ deviation relative to the Vienna Pee Dee Belemnite (VPDB). Mean values are shown with reproducibility tests that are better than ±0.05‰ for $\delta^{18}O$ and ±0.02‰ for $\delta^{13}C$ based on duplicate and triplicate measurements. Long-term analytical precision based on repeated measurements of an in-house marble carbonate standard verified against NBS-19 is shown in plot.

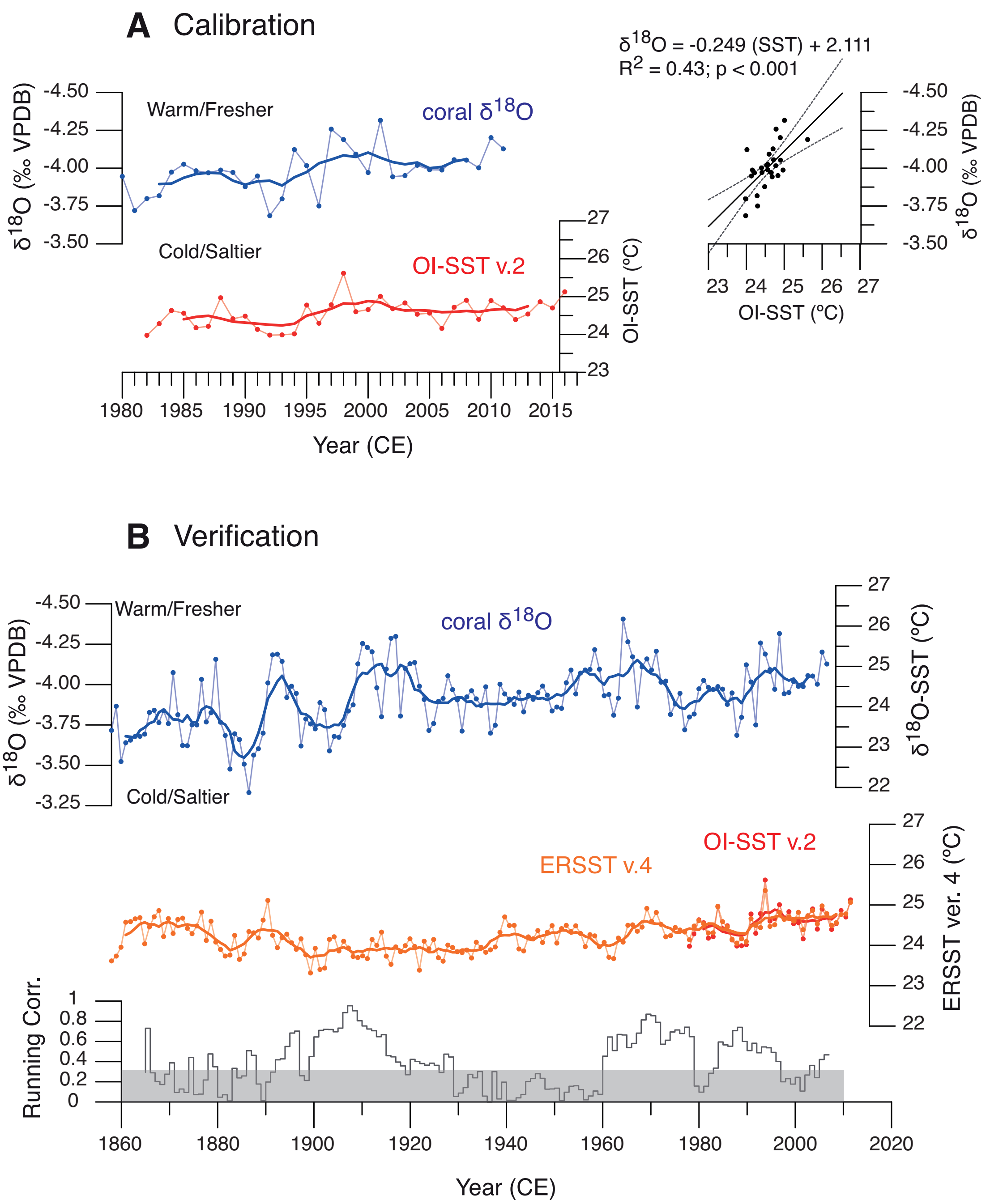
A Calibration
δ18O = -0.249 (SST) + 2.111
R2 = 0.43; p < 0.001
Warm/Fresher
coral δ18O
Cold/Saltier
OI-SST v.2
δ18O (‰ VPDB)
OI-SST (ºC)
Year (CE)
B Verification
ERSST v.4
δ18O-SST (ºC)
ERSST ver. 4 (ºC)
Running Corr.

Supplementary Fig. 6. Calibration and verification of coral δ^{18}O-SST proxy.

(a) Comparison between coral δ^{18}O (blue) and 4-grid mean NOAA NCEI Optimum Interpolation Sea Surface Temperature version 2 (OI-SST v.2; red) [2] over the period 1981-2011 CE. The 1º by 1º grids can be found on Supplementary Table 1. Calibration of annually-averaged δ^{18}O to the 4-grid OI-SST was completed by least squares linear regression with calibration equation shown. **(b)** Verification of coral-based δ^{18}O-SST (blue) comparison to 4-grid mean NOAA NCEI Extended Reconstructed SST version 4 (ERSST v.4; orange) [3] of the overlapping period, 1860-2011 CE. The 2º by 2º grids can be found on Supplementary Table 1. Superimposed on each individual time series are the 7-year running-means applied in the running correlation analysis of coherence between the coral δ^{18}O-SST and ERSST v.4 over the period 1860-2011 CE. The time period with the higher number of SST observations with highest level of confidence (Supplementary Fig. 7) yielded strong coherence. Below the shaded bar are insignificant coherence values ($p < 0.01$).

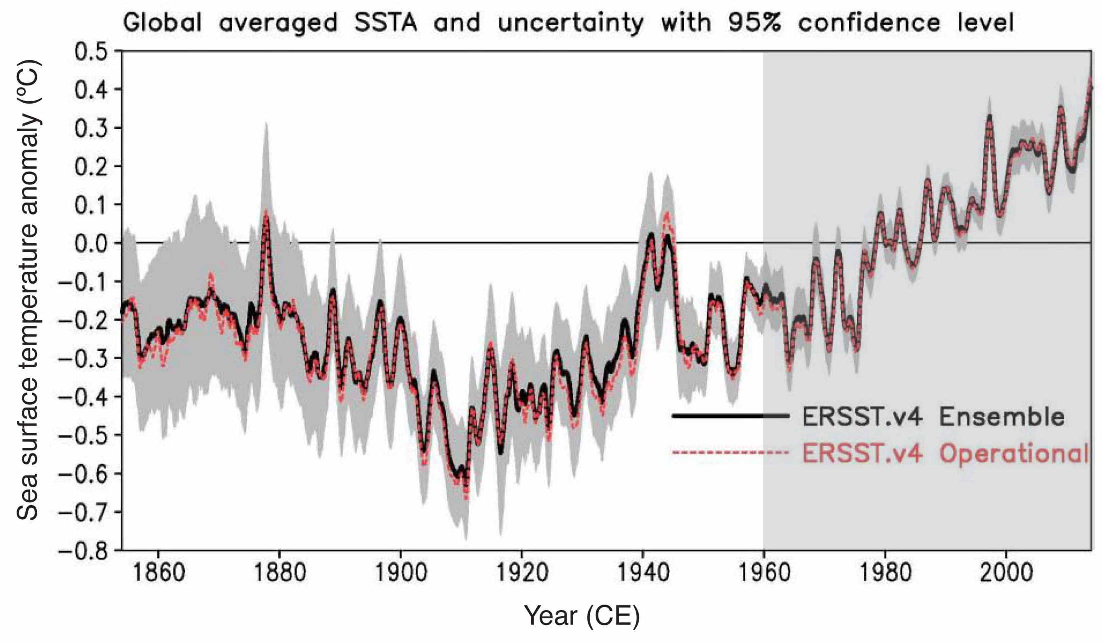

Supplementary Fig. 7. Extended Reconstructed Sea Surface Temperature version 4.

Global monthly averaged ERSST v. 4 anomaly [3] from 1854-2014 with uncertainty at 95% confidence level. Data reliability increases in the late twentieth century corresponding to increase in SST observations based on satellite observations (shaded grey). Image adapted from NOAA NCEI accessed from [https://www.ncdc.noaa.gov/data-access/marineocean-data/extended-reconstructed-sea-surface-temperature-ersst-v4].

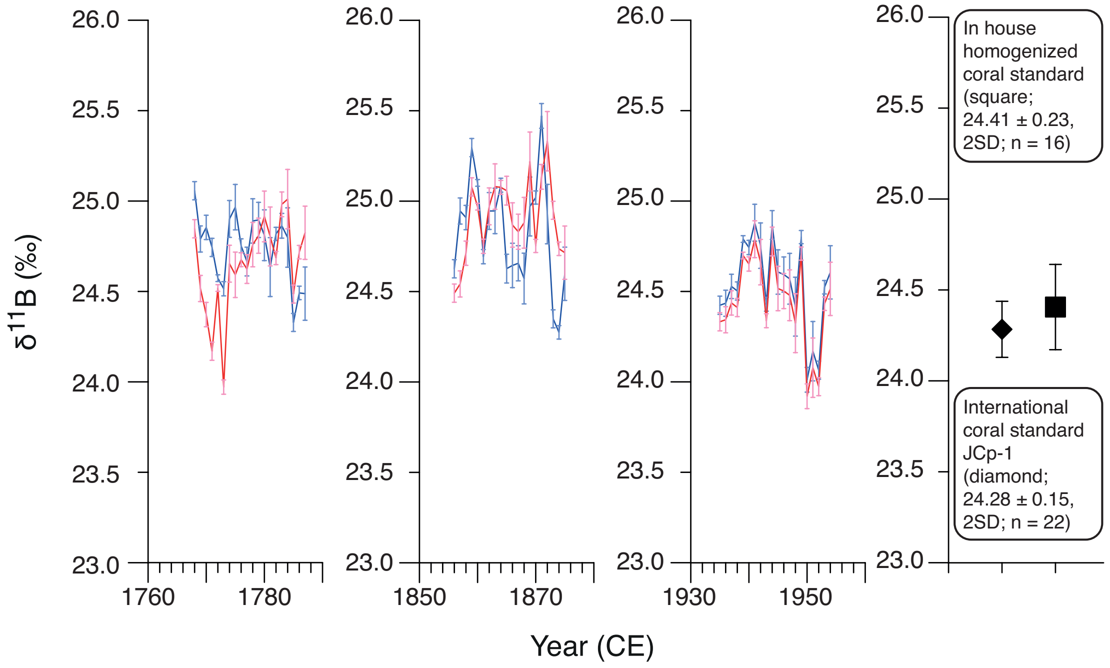

Supplementary Fig. 8. ***D. heliopora*** **intra-coral $\delta^{11}B$ ratio reproducibility.** The three replicated intra-colony coral $\delta^{11}B$ sections covering separate 20-year periods (1935-1954, 1856-1875, and 1768-1787 CE) are shown with the 2σ uncertainty of each individual measurement. The primary sampling track results are shown in red and the secondary sampling track results are in blue. Refer to Supplementary Fig. 2 for sampling transect locations. The analytical uncertainty of the international coral standard JCp-1 and the instrumental uncertainty of an in-house coral standard are reported in ‰ deviation relative to the NBS SRM 951 standard (boric acid isotopic standard).

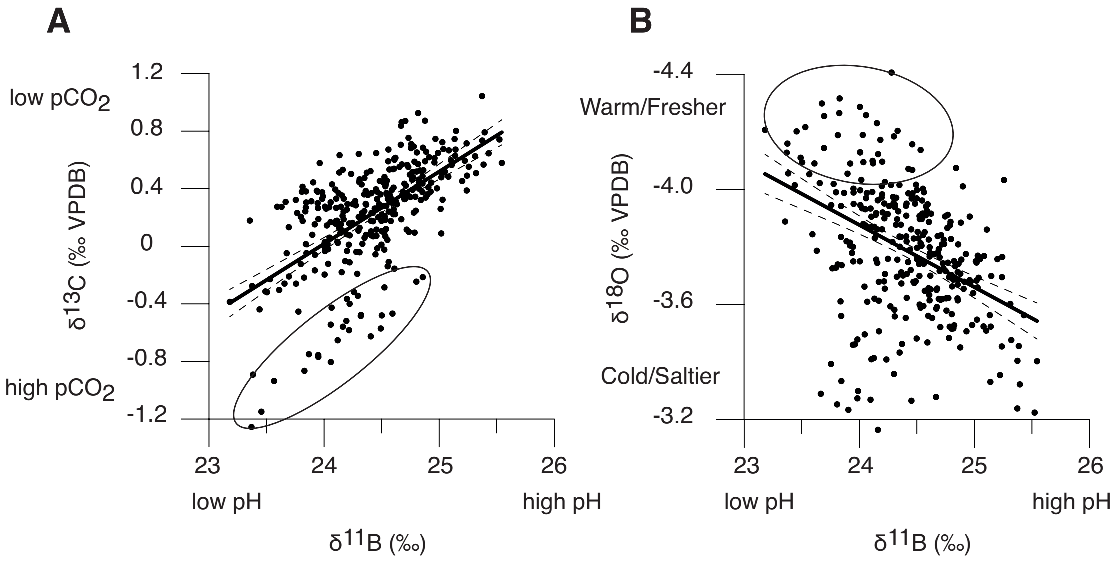

Supplementary Fig. 9. *D. heliopora* coral proxies correlation.

(**a**) Pearson's product-moment correlation analysis between the proxy records of $\delta^{11}B$ and $\delta^{13}C$ ($R = 0.61$, $p < 0.01$, $n = 319$). (**b**) Correlation analysis between the proxy records $\delta^{11}B$ and $\delta^{18}O$ ($R = 0.42$, $p < 0.01$, $n = 319$). Large zones situated outside of the main regression line areas are circled and represent the most recent ~30-year period.

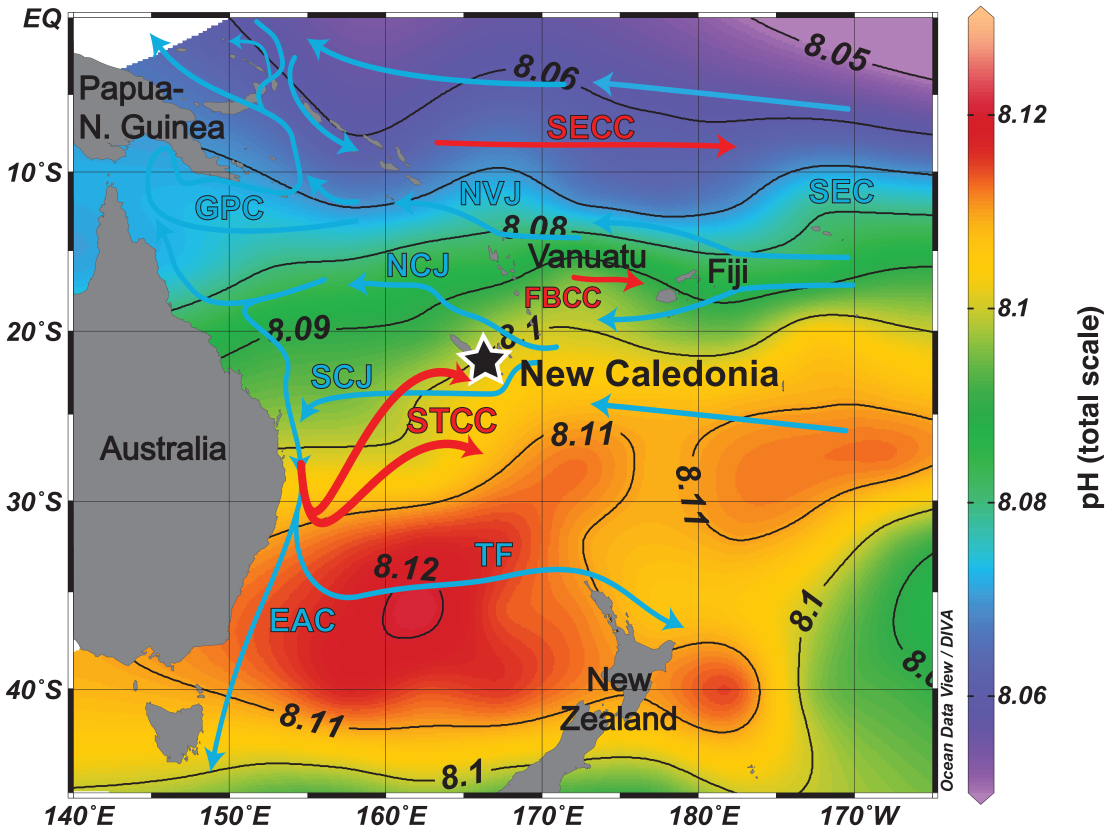

Supplementary Fig. 10. Major oceanic currents of Southwest Pacific. Annual gridded mean estimated seawater pH map [4] of the Southwest Pacific with major oceanic currents impacting the region near New Caledonia (star) modified from Figure 2 of ref. [5] (© 2014. American Geophysical Union. All Rights Reserved). Briefly, the blue arrows denote the main currents, integrated 0–1000 m (South Equatorial Current, SEC; North Vanuatu Jet, NVJ; Gulf of Papua Current, GPC; North Caledonian Jet, NCJ; South Caledonian Jet, SCJ; Tasman Front, TF; East Australia Current, EAC. The red arrows indicate the main surface-trapped counter-currents (South Pacific Subtropical Counter Current, STCC; Fiji Basin Counter Current, FBCC; and South Equatorial Counter Current, SECC). Map is produced using Ocean Data View ver. 4.7.4 (ref. [6]) (http://odv.awi.de) and modified manually.

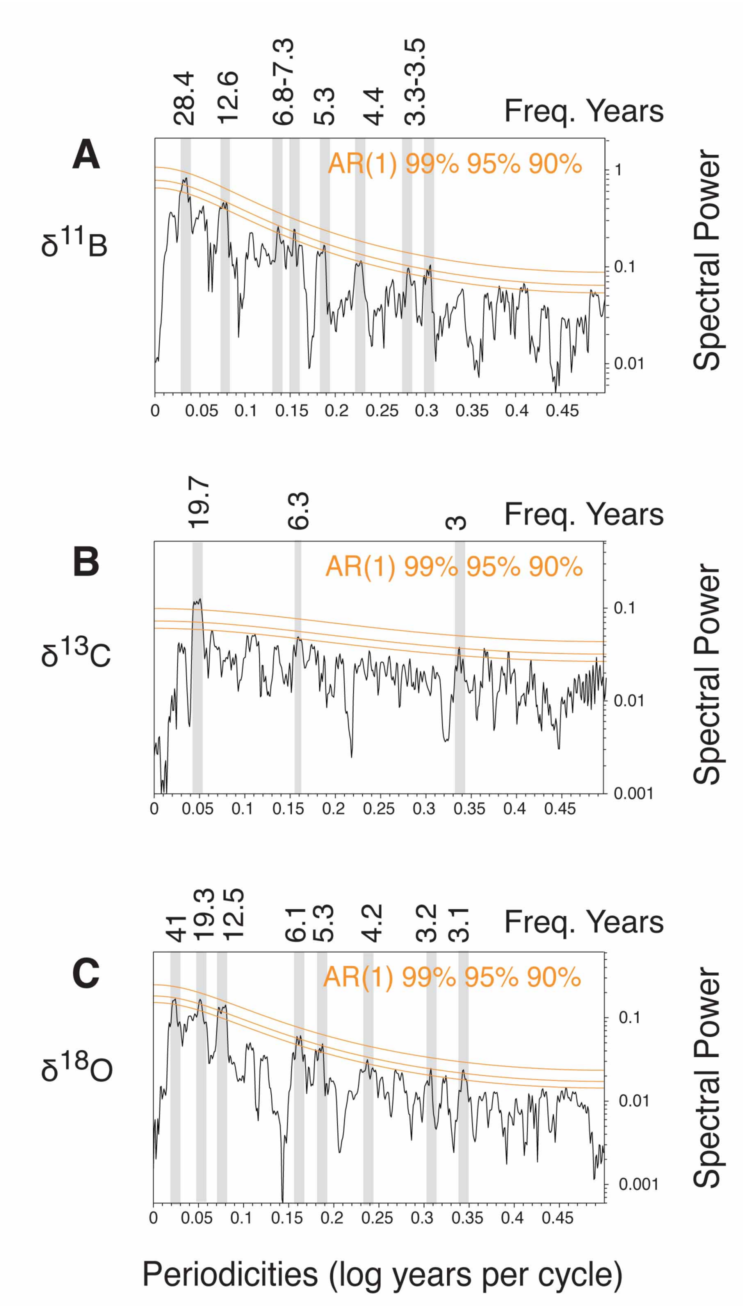

Supplementary Fig. 11. Singular Spectrum analysis results.
To estimate the power spectrum of each proxy time series record (**a**) $\delta^{11}B$ (‰), (**b**) $\delta^{13}C$ (‰ VPDB), and (**c**) $\delta^{18}O$ (‰ VPDB), Multi-Taper Method (MTM) spectral analysis [7] was completed on the detrended proxy records (Fig. 7). The power spectral density and significance was determined relative to a red noise null hypothesis [8] with the robust method of noise background estimation [8] using the configuration of 3 for tapers and 2 for resolution. Significant periodicities (log years per cycle) between 90, 95, and 99% confidence intervals are shown.

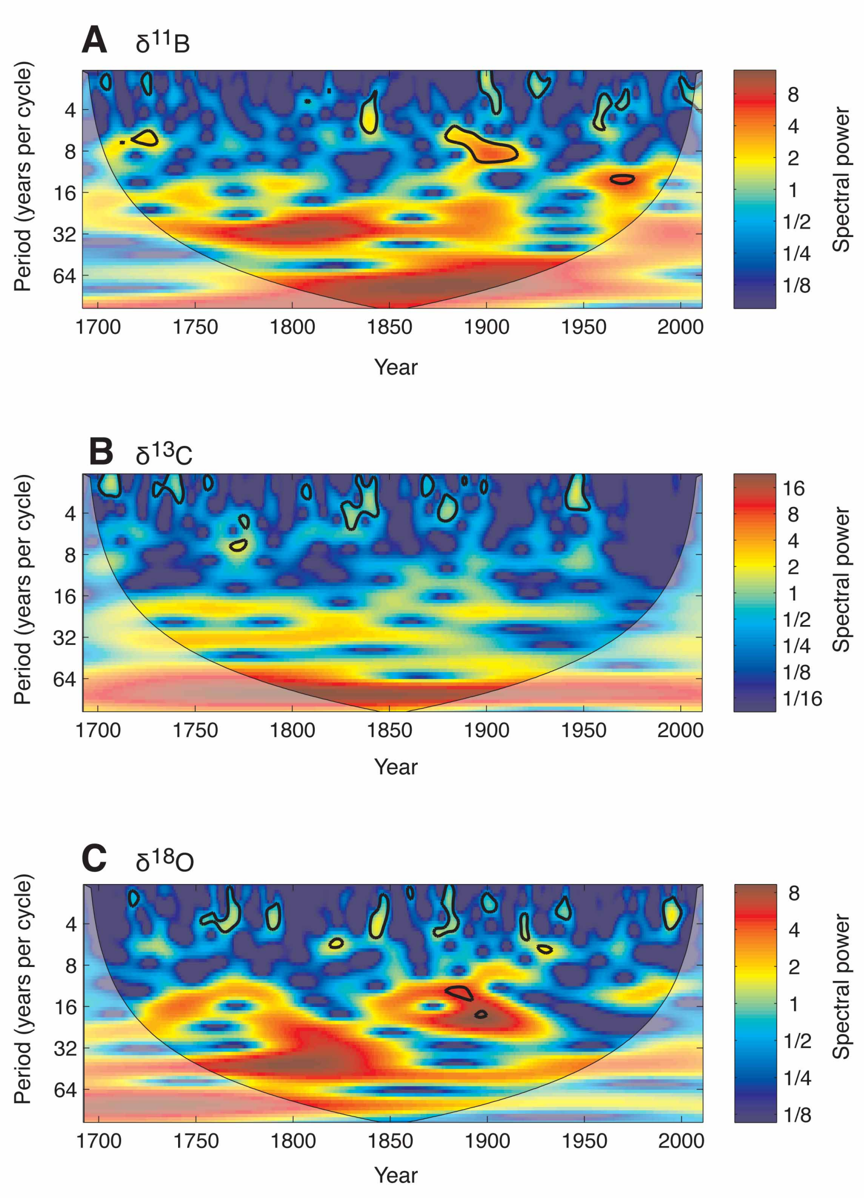

Supplementary Fig. 12. Coral proxies wavelet spectrum results. Wavelet spectrum plot [9] for each time series record: (**a**) $\delta^{11}B$ (‰), (**b**) $\delta^{13}C$ (‰ VPDB), and (**c**) $\delta^{18}O$ (‰ VPDB). The y-axis of each individual panel depicts the periodicity in years per cycle of the changing time series variability. The x-axis of each individual panel is the length of the time series record. The spectral power gradient is shown on the right side of each panel with increasing spectral power at the top (warmer colours equates to stronger spectral power). The black contours are the 10% significance regions, using a red-noise background spectrum.

Supplementary Tables.

Supplementary Table 1. Gridded Sea Surface Temperature calibration and verification.

4-grid (regional-scale) annual mean SST was compiled from the following individually gridded sea surface temperature (SST) datasets for calibration and verification of the *Diploastrea heliopora* coral skeletal $\delta^{18}O$-SST. The 1º by 1º gridded NOAA NCEI Optimum Interpolation SST version 2 (ref. [2]) from in-situ and satellite measurements spanned the period 1981-2011 CE and was used for calibration to the coral $\delta^{18}O$ time series. The 2º by 2º gridded NOAA NCEI Extended Reconstructed SST version 4 (ref. [3]) spanned the period 1860-2011 CE and was used for verification of the coral $\delta^{18}O$ time series.

	**$\delta^{18}O$-SST Calibration (1981-2011 CE)**		**$\delta^{18}O$-SST Verification (1860-2011 CE)**	
	**Optimum Interpolation SST ver. 2**		**Extended Reconstructed SST ver. 4**	
	**Lat.**	**Long.**	**Lat.**	**Long.**
**Grid 1**	22.5 S	165.5 E	22 S	166 E
**Grid 2**	22.5 S	166.5 E	22 S	164 E
**Grid 3**	23.5 S	165.5 E	24 S	166 E
**Grid 4**	23.5 S	166.5 E	24 S	164 E

Supplementary Table 2. Central and western Pacific coral $\delta^{13}C$ records. List of the published and publicly available coral $\delta^{13}C$ records described in Fig. 3 from the central and western Pacific Ocean (NOAA NCEI Paleoclimatology, https://www.ncdc.noaa.gov/data-access/paleoclimatology-data/datasets/coral-sclerosponge). As the published time series vary in their temporal resolution from monthly up to 5-years, the coral $\delta^{13}C$ records are downscaled and shown as smoothed 5-year moving mean. Due to inter-colony and inter-species offsets, removing the mean $\delta^{13}C$ value from each individual record over the twentieth century centres all coral $\delta^{13}C$ records. References for each record are listed with site coordinates, length of record, resolution, and coral species.

Study	Site location	Lat. & long.	Record length	Resolution	Coral species
[10]Bagnato et al., 2004	Savusavu Bay, Fiji	16.82S, 179.23E	1940 - 1997	Monthly	*Diploastrea heliopora*
[10]Bagnato et al., 2004	Savusavu Bay, Fiji	16.82S, 179.23E	1939 - 2001	Monthly	*Diploastrea heliopora*
[11]Boiseau et al., 1999	Moorea Lagoon, French Polynesia	17.5S, 149.83W	1852 - 1990	Annual	*Porites lutea*
[12]Cole et al., 1993	Tarawa Atoll, Kiribati	1N, 172E	1960 - 1977	Monthly	*Hydnophora microconos*
[12]Cole et al., 1993	Tarawa Atoll, Kiribati	1N, 172E	1959 - 1979	Monthly	*Hydnophora microconos*
[13]Dassié et al., 2013	Savusavu Bay, Fiji	16.82S, 179.23E	1781 - 1997	Monthly	*Porites* sp.
[14]Deng et al., 2013	Hainan Island, China	18.13N, 109.30E	1853 - 2011	Monthly & annual	*Porites lutea*
[15]Druffel et al., 1993	Abraham Reef, GBR, Australia	22.1S, 153E	1635 - 1957	2-year	*Porites australiensus*
[16]Evans et al., 1998	Kiritimati Island, Kiribati	2N, 157.3W	1938 - 1993	Monthly	*Porites* sp.
[17]Felis et al., 2009	Ogasawara Island, Japan	27.106N, 142.194E	1873 - 1994	Annual	*Porites* sp.
[18]Gorman et al., 2012	Sabine Bank, Vanuatu	15.94S, 166.04E	1842 - 2007	Monthly	*Porites lutea*
[19]Kilbourne et al., 2004	Malo Channel, Vanuatu	15.7S, 167.2E	1928 - 1992	Monthly	*Porites lutea*
[20]Liu et al., 2014	Hainan Island, China	18.12N, 109.29E	1838 - 2001	3-year	*Porites* sp.
[21]Osborne et al., 2014	Ulong Channel, Palau	7.28N, 134.25E	1793 - 2008	Monthly	*Porites* sp.
[21]Osborne et al., 2014	Rock Island, Palau	7.27N, 134.38E	1899 - 2008	Monthly	*Porites* sp.
[22]Pelejero et al., 2005	Flinders Reef, GBR, Australia	17.73S, 148.43E	1708 - 1988	5-year	*Porites* sp.
[23]Quinn & Sampson, 2002	Amédée Lighthouse, New Caledonia	22.48S, 166.47E	1968 - 1992	Monthly	*Porites lutea*
[24]Quinn et al., 1996	Espiritu Santo Island, Vanuatu	15S, 167E	1806 - 1979	Annual	*Platgyra lamellina*
[25]Quinn et al., 1998	Amédée Lighthouse, New Caledonia	22.29S, 166.27E	1657 - 1992	Monthly & seasonal	*Porites lutea*
[26]Schmidt et al., 2004	Guadalcanal, Solomon Islands	9.83S, 160.83E	1944 - 1994	Annual	*Porites* sp.
[27]Stephans et al., 2004	Amédée Lighthouse, New Caledonia	22.29S, 166.28E	1954 - 1999	Monthly	*Porites lutea*
[28]Wei et al., 2009	Arlington Reef, GBR, Australia	16.43S, 146.02E	1807 - 2004	5-year & annual	*Porites* sp.
**This study**	**Fausse Passe de Uitoé, New Caledonia**	**22.21S, 166.15E**	**1689 - 2011**	**Annual**	***Diploastrea heliopora***

Supplementary Table 3. Trends of central and western Pacific $\delta^{13}C$ decrease from 1978.

The mean secular trend of published and publicly available coral $\delta^{13}C$ records listed in Supplementary Table 2 from the central and western Pacific Ocean (from 1978 only) that match the atmospheric CO_2 station time series length as depicted in Fig. 3. As the coral time series vary in their temporal resolution and to assist in this secular trend comparison, the listed coral records were downscaled to annual averages. Linear trends over time of each $\delta^{13}C$ series spanning the period from 1978 to the most recent decades over its individual length were calculated. Some $\delta^{13}C$ series in Supplementary Table 2 were omitted from analysis due to record length that do not span the period from 1978 to the most recent decades. Presented on the bottom row is the western and central Pacific atmospheric CO_2 stations $\delta^{13}C$ time series from the Scripps CO_2 Program, Scripps Institution of Oceanography [29] (Fig. 3).

Study	Site location	Period from 1978 to	Change in $\delta^{13}C$ (‰ yr^{-1})	Coral species
[10]Bagnato et al., 2004	Savusavu Bay, Fiji	1997	-0.032	*Diploastrea heliopora*
[10]Bagnato et al., 2004	Savusavu Bay, Fiji	2001	-0.030	*Diploastrea heliopora*
[11]Boiseau et al., 1999	Moorea Lagoon, French Polynesia	1990	-0.018	*Porites lutea*
[13]Dassié et al., 2013	Savusavu Bay, Fiji	1997	-0.042	*Porites* sp.
[14]Deng et al., 2013	Hainan Island, China	2011	-0.002	*Porites lutea*
[16]Evans et al., 1998	Kiritimati Island, Kiribati	1993	-0.014	*Porites* sp.
[17]Felis et al., 2009	Ogasawara Island, Japan	1994	-0.054	*Porites* sp.
[18]Gorman et al., 2012	Sabine Bank, Vanuatu	2007	-0.026	*Porites lutea*
[19]Kilbourne et al., 2004	Malo Channel, Vanuatu	1992	-0.012	*Porites lutea*
[21]Osborne et al., 2014	Ulong Channel, Palau	2008	-0.010	*Porites* sp.
[21]Osborne et al., 2014	Rock Island, Palau	2008	-0.031	*Porites* sp.
[23]Quinn & Sampson, 2002	Amédée Lighthouse, New Caledonia	1992	-0.027	*Porites lutea*
[25]Quinn et al., 1998	Amédée Lighthouse, New Caledonia	1992	-0.028	*Porites lutea*
[26]Schmidt et al., 2004	Guadalcanal, Solomon Islands	1994	-0.019	*Porites* sp.
[27]Stephans et al., 2004	Amédée Lighthouse, New Caledonia	1999	-0.034	*Porites lutea*
[28]Wei et al., 2009	Arlington Reef, GBR, Australia	2004	-0.048	*Porites* sp.
**This Study**	**New Caledonia**	**2011**	**-0.024**	***Diploastrea heliopora***
Keeling et al., 2010*	SIO 5 Pacific Station mean*	2008*	-0.024*	*Atmospheric* CO_2*

* Data retrieved from Scripps Institution of Oceanography's Scripps CO_2 Program [29]. The stations used in the compilation including the time series span in brackets.

- Station Christmas Island, South Pacific (1978-2008)
- Station Kermadec Islands, South Pacific (1985-2008)
- Station Kumukahi, Hawaii (1981-2008)
- Station Mauna Loa, Hawaii (1981-2008)
- Station American Samoa, South Pacific (1985-2008)

Supplementary Table 4. List of major volcanic eruptions since 1600 CE. Major volcanic eruptions of approximately magnitude 6 or larger on the Volcanic Explosivity Index (VEI) since 1600 CE. Volcanic events in Fig. 3 describe only tropical Pacific forcing and are partially listed in this table. This is not an exhaustive list of all volcanic activity. List of volcanic eruptions partially compiled from ref. [30,31].

* Unattributed volcanic events are marked as (UE)

Name and area	Year	Volcanic Explosivity Index (VEI)
Pinatubo, Philippines	1991	6
Mt. St. Helens, Washington, USA	1980	5
Novarupta, Alaska, USA	1912	6
Santa Maria, Guatemala	1902	6
Mt. Tarawera, New Zealand	1886	5
Krakatoa, Indonesia	1883	6
Mt. Tambora, Indonesia	1815	7
UE 1809	1809	NA
Grímsvötn, Iceland	1784	6
Laki, Iceland	1783	6
UE 1695	1695	NA
Long Island, Papua New Guinea	1660	6
Kolumbo, Santorini, Greece	1650	6
Parker, Philippines	1641	6
Huaynaputina, Peru	1601	6

Supplementary Table 5. Western and southwestern Pacific coral $\delta^{11}B$ records.

The published and publicly available coral $\delta^{11}B$ records from the western and southwestern Pacific shown in Fig. 4. References for each record are listed with site coordinates, length of record, resolution, coral species, and analytical methodology. All records listed here and shown in Fig. 4 are in the native temporal resolution as the original publication. The reconstructed seawater pH in Fig. 4 plotted as secondary y-axis are reproduced from the original citation and were not converted based on our methodology (see Methods).

Publication	Coral location	Lat. & long.	Coral species	Record length	Resolution	$\delta^{11}B$ ‰ (Min. - Max.)
[32]D'Olivo et al., 2015	Pandora Reef, GBR, Australia	18.82S, 146.43E	*Porites* sp.	1963 - 2002	Annual	23.04 – 25.95 †
[32]D'Olivo et al., 2015	Havannah Island, GBR, Australia	18.85S, 146.55E	*Porites* sp.	1999 - 2009	Annual	23.34 – 24.43 ‡
[32]D'Olivo et al., 2015	Havannah Island, GBR, Australia	18.85S, 146.55E	*Porites* sp.	1940 - 2009	Annual	22.49 – 25.26 ‡
[32]D'Olivo et al., 2015	Havannah Island, GBR, Australia	18.85S, 146.55E	*Porites* sp.	1966 - 2005	Annual	24.26 – 26.27 †
[32]D'Olivo et al., 2015	Rib Reef, GBR, Australia	18.48S, 146.88E	*Porites* sp.	1961 - 2009	Annual	23.11 – 25.41 †
[32]D'Olivo et al., 2015	17-065 Reef, GBR, Australia	18.05S, 146.98E	*Porites* sp.	1973 - 2009	Annual	22.52 – 24.13 ‡
[20]Liu et al., 2014	Hainan Island, China	18.12N, 109.29E	*Porites* sp.	1838 - 2001	3-year	22.16 – 24.91 #
[22]Pelejero et al., 2005	Flinders Reef, GBR, Australia	17.73S, 148.43E	*Porites* sp.	1708 - 1988	5-year	22.99 – 24.97 †
[33]Shinjo et al., 2013	Guam	13.6N, 144.8E	*Porites* sp.	1940 - 1999	Annual	22.10 – 23.92 #
[28]Wei et al., 2009	Arlington Reef, GBR, Australia	16.43S, 146.02E	*Porites* sp.	1807 - 2004	5-year	21.06 – 25.38 †
[34]Wei et al., 2015	Hainan Island, China	18.13N, 109.30E	*Porites lutea*	1853 - 2011	Monthly & annual	20.82 – 26.00 *
**This study**	**Fausse Passe de Uitoé, New Caledonia**	**22.21S, 166.15E**	***D. heliopora***	**1689 - 2011**	**Annual**	**23.18 – 25.54 ***

* Thermo Scientific Neptune Plus Multi-Collector Inductively Coupled Plasma Mass Spectrometer (MC-ICP-MS)
† Thermo Scientific TRITON Thermal Ionization Mass Spectrometer (TIMS)
‡ NU Plasma II MC-ICP-MS
\# Thermo Scientific Neptune MC-ICP-MS

Supplementary References

1. Ryan, W. B. F. *et al.* Global Multi-Resolution Topography synthesis. *Geochemistry, Geophys. Geosystems* **10,** Q03014 (2009).
2. Smith, T. M., Reynolds, R. W., Peterson, T. C. & Lawrimore, J. Improvements to NOAA's Historical Merged Land–Ocean Surface Temperature Analysis (1880–2006). *J. Clim.* **21,** 2283–2296 (2008).
3. Huang, B. *et al.* Extended Reconstructed Sea Surface Temperature Version 4 (ERSST.v4). Part I: Upgrades and Intercomparisons. *J. Clim.* **28,** 911–930 (2014).
4. Takahashi, T. *et al.* Climatological distributions of pH, pCO_2, total CO_2, alkalinity, and $CaCO_3$ saturation in the global surface ocean, and temporal changes at selected locations. *Mar. Chem.* **164,** 95–125 (2014).
5. Ganachaud, A. *et al.* The Southwest Pacific Ocean circulation and climate experiment (SPICE). *J. Geophys. Res. Ocean.* **119,** 7660–7686 (2014). John Wiley & Sons Ltd.
6. Schlitzer, R. Ocean Data View. (2015). at <http://odv.awi.de>
7. Ghil, M. *et al.* Advanced spectral methods for climatic time series. *Rev. Geophys.* **40,** 1–41 (2002).
8. Mann, M. E. & Lees, J. M. Robust estimation of background noise and signal detection in climatic time series. *Clim. Change* **33,** 409–445 (1996).
9. Torrence, C. & Compo, G. A practical guide to wavelet analysis. *Bull. Am. Meteorol. Soc.* **79,** 61–78 (1998).
10. Bagnato, S., Linsley, B. K., Howe, S. S., Wellington, G. M. & Salinger, J. Evaluating the use of the massive coral *Diploastrea heliopora* for paleoclimate reconstruction. *Paleoceanography* **19,** PA1032 (2004).
11. Boiseau, M., Ghil, M. & Juillet-Leclerc, A. Climatic trends and interdecadal variability from south-central pacific coral records. *Geophys. Res. Lett.* **26,** 2881–2884 (1999).
12. Cole, J. E., Fairbanks, R. G. & Shen, G. T. Recent variability in the southern oscillation: isotopic results from a Tarawa Atoll coral. *Science* **260,** 1790–1793 (1993).
13. Dassié, E. P., Lemley, G. M. & Linsley, B. K. The Suess effect in Fiji coral $\delta^{13}C$ and its potential as a tracer of anthropogenic CO_2 uptake. *Palaeogeogr. Palaeoclimatol. Palaeoecol.* **370,** 30–40 (2013).
14. Deng, W. *et al.* Variations in the Pacific Decadal Oscillation since 1853 in a coral record from the northern South China Sea. *J. Geophys. Res. Ocean.* **118,** 2358–2366 (2013).
15. Druffel, E. R. M. & Griffin, S. Large variations of surface ocean radiocarbon: Evidence of circulation changes in the southwestern Pacific. *J. Geophys. Res. Ocean.* **98,** 20249–20259 (1993).
16. Evans, M., Fairbanks, R. & Rubenstone, J. A proxy index of ENSO teleconnections. *Nature* **394,** 732–733 (1998).
17. Felis, T. *et al.* Subtropical coral reveals abrupt early-twentieth-century freshening in the western North Pacific Ocean. *Geology* **37,** 527–530 (2009).
18. Gorman, M. K. *et al.* A coral-based reconstruction of sea surface salinity at Sabine Bank, Vanuatu from 1842 to 2007 CE. *Paleoceanography* **27,**

PA3226 (2012).
19. Kilbourne, K. H., Quinn, T. M., Taylor, F. W., Delcroix, T. & Gouriou, Y. El Niño–Southern Oscillation–related salinity variations recorded in the skeletal geochemistry of a *Porites* coral from Espiritu Santo, Vanuatu. *Paleoceanography* **19,** 1–8 (2004).
20. Liu, Y. *et al.* Acceleration of modern acidification in the South China Sea driven by anthropogenic CO_2. *Sci. Rep.* **4,** 5148 (2014).
21. Osborne, M. C., Dunbar, R. B., Mucciarone, D. A., Druffel, E. & Sanchez-Cabeza, J.-A. A 215-yr coral $\delta^{18}O$ time series from Palau records dynamics of the West Pacific Warm Pool following the end of the Little Ice Age. *Coral Reefs* **33,** 719–731 (2014).
22. Pelejero, C. *et al.* Preindustrial to modern interdecadal variability in coral reef pH. *Science* **309,** 2204–2207 (2005).
23. Quinn, T. M. & Simpson, D. E. A multiproxy approach to reconstructing sea surface conditions using coral skeleton geochemistry. *Paleoceanography* **17,** 1062 (2002).
24. Quinn, T. M., Crowley, T. J. & Taylor, F. W. New stable isotope results from a 173-year coral from Espiritu Santo, Vanuatu. *Geophys. Res. Lett.* **23,** 3413–3416 (1996).
25. Quinn, T. M. *et al.* A multicentury stable isotope record from a New Caledonia coral: Interannual and decadal sea surface temperature variability in the southwest Pacific since 1657 A.D. *Paleoceanography* **13,** 412–426 (1998).
26. Schmidt, A., Burr, G. S., Taylor, F. W., O'Malley, J. & Beck, J. W. A semiannual radiocarbon record of a modern coral from the Solomon Islands. *Nucl. Instruments Methods Phys. Res. Sect. B Beam Interact. with Mater. Atoms* **223–224,** 420–427 (2004).
27. Stephans, C. L. Assessing the reproducibility of coral-based climate records. *Geophys. Res. Lett.* **31,** 2–5 (2004).
28. Wei, G., McCulloch, M. T., Mortimer, G., Deng, W. & Xie, L. Evidence for ocean acidification in the Great Barrier Reef of Australia. *Geochim. Cosmochim. Acta* **73,** 2332–2346 (2009).
29. Keeling, R. F., Piper, S. C., Bollenbacher, A. F. & Walker., S. J. Monthly atmospheric $^{13}C/^{12}C$ isotopic ratios for 11 SIO stations. *Trends: A Compendium of Data on Global Change* (2010).
30. Sigl, M. *et al.* Timing and climate forcing of volcanic eruptions for the past 2,500 years. *Nature* **523,** 543–549 (2015).
31. Global Volcanism Program (Smithsonian Institution). Eruptions, Earthquakes & Emissions, v.1. (2016). at <http://volcano.si.edu/E3/>
32. D'Olivo, J. P., McCulloch, M. T., Eggins, S. M. & Trotter, J. Coral records of reef-water pH across the central Great Barrier Reef, Australia: assessing the influence of river runoff on inshore reefs. *Biogeosciences* **12,** 1223–1236 (2015).
33. Shinjo, R., Asami, R., Huang, K.-F., You, C.-F. & Iryu, Y. Ocean acidification trend in the tropical North Pacific since the mid-20th century reconstructed from a coral archive. *Mar. Geol.* **342,** 58–64 (2013).
34. Wei, G. *et al.* Decadal variability in seawater pH in the West Pacific : Evidence from coral $\delta^{11}B$ records. *J. Geophys. Res. Ocean.* **120,** 7166–7181 (2015).
